# Morphometric and Molecular Insights into *Hepatozoon* spp. in Wild and Synanthropic Rodents from Southern and Southeastern Brazil

**DOI:** 10.3390/pathogens14080756

**Published:** 2025-07-31

**Authors:** Tatiana Pádua Tavares de Freitas, Bernardo Rodrigues Teixeira, Eduarda de Oliveira Silva Lima Machado, Isaac Leandro Lira Pinto, Laís da Silva de Oliveira, Karina Varella, Huarrisson Azevedo Santos, Fernando de Oliveira Santos, Liliani Marilia Tiepolo, Carlos Luiz Massard, Maristela Peckle

**Affiliations:** 1 Department of Animal Parasitology, Veterinary Institute, Federal Rural University of Rio de Janeiro-UFRRJ, Seropédica 23890-000, RJ, Brazil; tat.padua@gmail.com (T.P.T.d.F.); eduardamachadovet@gmail.com (E.d.O.S.L.M.); isaac.ufrrj@gmail.com (I.L.L.P.); sialais98@gmail.com (L.d.S.d.O.); carlosmassard@ufrrj.br (C.L.M.); maristelapeckle@ufrrj.br (M.P.); 2 Laboratory of Biology and Parasitology of Reservoir Wild Mammals (LABPMR), Oswaldo Cruz Institute-FIOCRUZ, Rio de Janeiro 21040-900, RJ, Brazil; karinavarella@gmail.com (K.V.); fernando.oliveira.snts@gmail.com (F.d.O.S.); 3 Department of Epidemiology and Public Health, Veterinary Institute, Federal Rural University of Rio de Janeiro-UFRRJ, Seropédica 23890-000, RJ, Brazil; huarrissonazevedo9@gmail.com; 4 Laboratory of Analysis and Monitoring of Atlantic Forest, Federal University of Paraná, Campus Litoral, Matinhos 83260-000, PR, Brazil; liliani@ufpr.br

**Keywords:** neotropical rodents, hemoparasites, 18S ribosomal DNA, haplotype diversity

## Abstract

Small rodents are known hosts of various pathogens, including *Hepatozoon*, but until now, in Brazil, only *Hepatozoon milleri* has been described in these animals. In this study, liver samples and blood smears were obtained from 289 rodents belonging to 14 Cricetidae and two Muridae species that had been captured in municipalities of the states of Paraná and Rio de Janeiro. Smears were stained with Giemsa, and gametocytes were detected via microscopy in 10.72% (*n* = 31/289) of samples, with these individuals representing three rodent species. Significant morphometric differences were observed in gametocyte measurements in *Akodon* rodents. Using conventional PCR, *Hepatozoon* spp. 18S rDNA fragments were amplified in 24.91% (*n* = 72/289) of samples, with those individuals representing seven rodent species. Phylogenetic analyses clustered 41 sequences from this study into a subclade with other sequences from small mammals in Brazil, identifying four distinct haplotypes, and, for the first time, a relationship between *Hepatozoon* haplotype and gametocyte length was observed. Based on phylogenetic analysis, this study reinforces the trophic relationship between rodents and reptiles as a possible link in the *Hepatozoon* transmission cycle in South America. Furthermore, our findings expand knowledge on *Hepatozoon* spp. hosts, describing *Oxymycterus nasutus* and *Oxymycterus quaestor* as new host species and identifying two novel circulating haplotypes in rodents from Paraná State, southern Brazil.

## 1. Introduction

Since the early 20th century, apicomplexan parasites of the genus *Hepatozoon* (Miller, 1908) (Adeleorina, Hepatozoidae) have been identified in a wide range of hosts, including rodents [1]. *Hepatozoon* are obligate heteroxenous parasites that infect mammals, reptiles, and amphibians, which serve as their intermediate hosts. In these vertebrate hosts, the parasites undergo merogony and the formation of gametocytes. In contrast, sexual reproduction and sporogony take place within hematophagous invertebrate hosts, such as ticks, mites, sand flies, tsetse flies, mosquitoes, fleas, lice, reduviid bugs, and leeches [2,3].

Transmission occurs through the ingestion of infected hematophagous ectoparasites [2,4]. Within the intestine of the arthropod vector, the ingested gametocytes undergo sexual reproduction, resulting in the formation of a zygote, which crosses the intestinal wall and migrates to the hemocoel, where it develops into a polysporocystic oocyst containing sporozoites. Infection of the vertebrate host occurs when the host ingests the infected arthropod, releasing the sporozoites and allowing the parasitic cycle to continue within its tissues [1,2].

In vertebrate hosts, vermicules penetrate membranes to reach the circulation and organs, where merogony occurs. Merozoites disseminate through the bloodstream, remaining in a quiescent stage (cystic stage) and invading leukocytes, where they differentiate into gametocytes [2,5,6]. The ingestion of ectoparasites is associated with the grooming behavior observed in mammals and plays a crucial role in the trophic transmission of certain parasites [7]. It has also been suggested that transmission may occur through the predation of vertebrates acting as paratenic intermediate hosts [2,8,9,10], and there are reports of vertical transmission in dogs [11] and rodents [12].

Wild rodents infected with *Hepatozoon* spp. have been reported in various regions of the world, including South America, with records in Brazil [13,14,15,16,17,18,19] and Chile [20,21,22]. It has been suggested that they play a role as paratenic hosts of *Hepatozoon* spp. due to the presence of parasitic cysts in their tissues and their participation in the trophic network as prey [6,10,23]. Few studies have described health alterations in rodents infected with *Hepatozoon* spp. Miller (1908) observed that rodents with massive infections exhibited anemia, which could result in the animals’ deaths [1]. More recently, in a study of rodents naturally infected with *Hepatozoon* sp. in Pakistan, a significant increase in oxidative stress markers was detected in vital organs (heart, kidney, lung, and liver), suggesting tissue damage, along with alterations in hematological parameters [24].

In Brazil, the first report of infection by this parasite in wild rodents, the morphological description of *Hepatozoon muris* in *Akodon fuliginosus* (=*Thaptomys nigrita*), dates back to 1915 and originated from the state of São Paulo [25]. More recently, the first molecular detection of *Hepatozoon* sp. infection in the rodent species *Calomys callosus* was recorded in the state of Mato Grosso [14], and a new parasite species, *Hepatozoon milleri*, was described in *Akodon montensis* rodents in São Paulo [6]. Other reports of molecular detection of *Hepatozoon* spp. in small wild rodents have been recorded in the states of Bahia [17], Mato Grosso [17], Mato Grosso do Sul [16,17,18,19], São Paulo [6,19], and Rio de Janeiro [17]. Although Demoner et al. [6] described a new *Hepatozoon* species parasitizing small rodents, most studies on wild and synanthropic rodents do not identify the parasite at the species level, suggesting that there may be a high diversity of *Hepatozoon* species within this mammalian population.

This study aimed to expand knowledge on *Hepatozoon* spp. in wild and synanthropic rodents captured in municipalities of the southern and southeastern regions of Brazil, integrating gametocyte morphometric analysis, genetic-diversity analysis, and phylogenetic analysis for species characterization.

## 2. Materials and Methods

### 2.1. Rodent Capture and Handling

In order to capture the rodents used in this study, Sherman^®^ (H.B. Sherman Traps, Inc., Tallahassee, FL, USA) and Tomahawk^®^ (Tomahawk Live Trap Co., Tomahawk, WI, USA) traps were set up over a period of four to six consecutive nights in the municipalities of Ponta Grossa (PG: 25°11′58.82″ S, 49°57′10.33″ W), Cruz Machado (CM: 26°4′25.7″ S, 51°25′0.5″ W), and Lidianópolis (LD: 24°8′15.02″ S, 51°38′25.60″ W) in the state of Paraná, as well as in Nova Friburgo (NF: 22°13′23″ S, 42◦38′16″ W) and Iguaba Grande (IG: 22°50′21.01″ S, 42°13′44.00″ W) in the state of Rio de Janeiro, all of which are situated in the southern and southeastern regions of Brazil.

The study areas in the southern region (Paraná State) are located within the Atlantic Forest biome, with *Araucaria angustifolia* pine tree vegetation (mixed ombrophilous forest) in CM and *A. angustifolia* vegetation with natural grasslands in PG [26]. The municipality of LD is located in the seasonal semideciduous forest region, and all three study areas have mesothermal temperate subtropical climates [26]. NF is situated in the mountainous region of the Atlantic Forest in Rio de Janeiro, which is characterized by a high-altitude tropical climate [27]. In contrast, IG is located in a region called “Região dos Lagos,” where rodent capture took place in vegetation typical of the restinga ecosystem, along the coastal area of the Atlantic Forest biome.

The expeditions took place between 2019 and 2022. In NF, four expeditions were conducted during this period, whereas in the other municipalities, only one expedition was carried out (Table 1). The traps were inspected and baited daily with a mixture of banana, oats, peanuts, and bacon, and captured specimens were transferred to a field laboratory categorized as biosafety level 3 [28,29]. All procedures were conducted on humanely euthanized rodents in accordance with the experimental protocol approved by the Ethics Committee for Animal Use at the Oswaldo Cruz Institute (L-036/2018). Blood and liver samples were collected to prepare blood smears and conduct molecular analysis, respectively. Subsequently, the rodents were taxidermized, identified through morphological, cytogenetic, and molecular analyses, and deposited in the Collection for Reservoir Wild Mammals (COLMASTO) at the Oswaldo Cruz Institute—FIOCRUZ-RJ [30]. The collection of small mammals was authorized by SISBIO-ICMBIO through licenses 13373 and 63846.

### 2.2. Preparation of Blood Smears for Gametocytes Research and Analysis

Two blood smears were prepared per animal, air-dried, and fixed in absolute methanol for 10 min. The slides were then stained with Giemsa in a 1:10 dilution and examined under an Olympus BX-51 optical microscope (Olympus Corporation, Tokyo, Japan) at a 1000× magnification.

For the morphometric analysis, cellSens Version Standard 2.1 software (Olympus Corporation, Tokyo, Japan) was used to measure the total lengths and widths of the gametocytes and their nuclei [6]. Individual averages, the overall mean, and the standard deviation were calculated for each parameter.

### 2.3. Statistical Analysis

The length and width of 10 gametocytes and their respective nuclei were measured for each host. These data were organized into tables for descriptive analysis, including the calculation of arithmetic means and standard deviations, and the normality of the data was assessed using the Lilliefors test.

To evaluate potential morphometric differences in gametocytes among predefined groups (such as rodent species, localities, and haplotypes), Tukey’s test and the Kruskal–Wallis test were applied, with all statistical analyses performed using BioEstat 5.3 software (Instituto Mamirauá, Belém, Brazil) [31].

### 2.4. DNA Extraction

Liver samples were preserved in RNAlater^®^ (Thermo Fisher Scientific, Waltham, MA, USA) and subsequently stored at −80 °C until DNA extraction. Genomic DNA was extracted from 25 mg liver samples from rodents using a DNeasy Blood and Tissue Kit (Qiagen, Hilden, Germany), following the manufacturer’s recommended protocol. The extracted DNA samples were then quantified using a Nanodrop^®^ spectrophotometer (Thermo Fisher Scientific Inc., Wilmington, MA, USA) and standardized to a concentration of 60 ng/μL.

### 2.5. Molecular Analysis

To confirm the DNA extraction, a fragment of the mammalian CytB gene (~800 bp) was amplified from each sample using polymerase chain reaction (PCR) with the primers MVZ05 and MVZ16 [32,33].

To detect the presence of *Hepatozoon* spp. DNA, a modified protocol developed by Ujvari et al. [34] was used; this protocol involves amplification of a fragment of the 18 SrDNA (~600 bp) through conventional PCR. The total PCR volume was 25 μL, with that volume containing 1U Platinum^®^ Taq DNA Polymerase (Invitrogen™, Thermo Fisher Scientific, Waltham, MA, USA), 1.5 mM MgCl2, 1× Buffer, 10 pmol of each primer (HepF300—5′ GTTTCTGACCTATCAGCTTTCGACG 3′ and Hep900—5′ CAAATCTAAGAATTTCACCTCTGAC 3′), and 0.4 mM dNTPs. The thermal cycling conditions were 94 °C for 4 min, followed by 35 cycles of 94 °C for 30 s, 55 °C for 30 s, and 72 °C for one minute, with a final extension at 72 °C for 7 min. Each PCR reaction used 3 μL of DNA. Negative controls (ultrapure water) and positive controls from a *Hepatozoon canis*-infected dog, with gametocytes visualized on a stained blood smear slide, were included in each reaction.

Subsequently, to obtain larger fragments of the 18S rDNA (~1600 bp), DNA samples that had tested positive in the previous reaction, representing each rodent species by locality, were subjected to a nested PCR (nPCR) reaction. For the first reaction (PCR), the primers HAM 1F (5′ GCCAGTAGTCATATGCTTGTC 3′) and HPF 2R (5′ GACTTCTCCTTCGTCTAAG 3′) [35] were used, and for the second reaction (nPCR), the primers EF-M (5′ AAAACTGCAAATGGCTCATT 3′) [36,37] and Hep1615R (5′ AAAGGGCAGGGACGTAATC 3′) [38] were employed, using a Promega® Master Mix kit (Madison, WI, USA). Each reaction consisted of 1× Master Mix (Taq DNA polymerase 50 units/mL, dATP 400 μM, dGTP 400 μM, dCTP 400 μM, dTTP 400 μM, MgCl_2_ 3 mM), 10 pmol of each primer, 3 μL of DNA (PCR) or 1 μL of the amplified product (nPCR), and ultrapure water to complete the final volume of 25 μL. The cycling parameters were 95 °C for 5 min, followed by 40 cycles of 95 °C for 45 s, 52 °C (PCR) or 55 °C (nPCR) for 45 s, 72 °C for 1 min, and a final extension at 72 °C for 7 min.

### 2.6. Electrophoresis of Reactions, Purification of Amplified Products, and Sequencing

The PCR products were subjected to electrophoresis on a 1.5% agarose gel with GelRed Nucleic Acid Stain (Biotium, Hayward, CA, USA) for one hour and observed under ultraviolet light using a transilluminator. The amplified products were purified using an Illustra GFX kit (Cytiva, Marlborough, MA, USA), and the samples were prepared for sequencing using a BigDye Terminator Cycle Sequencing Ready Reaction Kit v. 3.1 (Applied Biosystems, Foster, CA, USA) on an ABI3730xl DNA Analyzer (Applied Biosystems™) by capillary electrophoresis (SANGER) at the Fiocruz Technology Platforms Network RPT01A (Rio de Janeiro, Brazil).

### 2.7. Phylogenetic Analysis

The sequences from this study were assembled and edited using Geneious Prime 2025.0.3 (Biomatters Ltd., Auckland, New Zealand) by comparison with homologous sequences through the BLASTn tool (https://blast.ncbi.nlm.nih.gov/Blast.cgi accessed on 26 September 2024) [39]. After analysis, they were deposited in GenBank. The consensus sequences generated, including those from this study (PQ807526-66) and others retrieved from GenBank, were aligned using the Clustal W method in MEGA 11 (Pennsylvania State University, State College, PA, USA) [40] and trimmed to obtain two phylogenetic trees: one created using short sequences (>500 bp) and another created using long sequences (>1600 bp). A database was created with *Hepatozoon* spp. sequences selected according to the following criteria: the included sequences were (a) obtained from vertebrates, (b) from the same region of the 18S rDNA gene as the sequences in this study, and (c) of a size greater than 500 bp. Additionally, sequences obtained from GenBank of other members of the suborder Adeleorina, namely *Hemogregarina* spp., *Karyolysus paradoxa*, and *Dactylosoma* spp. (outgroup), were added to the database (Table S1).

For the Bayesian inference (BI), in both phylogenetic analyses, a nexus file was created from the alignment in the software Mesquite 3.81 (Mesquite Project, University of California, Davis, CA, USA) [41] for MrBayes 3.2.7 (University of Oslo, Oslo, Norway) [42] using the Markov Chain Monte Carlo (MCMC) method with the GTR + I + G model for 10,000,000 generations. BI phylogenetic reconstructions were performed using the MrBayes program, which was executed on the CIPRES Science Gateway platform (San Diego Supercomputer Center, University of California San Diego, La Jolla, CA, USA) [43]. The trees were summarized with a 25% burn-in discarded, and the effective sample size (ESS) of the MCMCs was analyzed using Tracer 1.7.2 (Andrew Rambaut, University of Edinburgh, Edinburgh, UK) [44]. The consensus trees were visualized in FigTree 1.4.1 (Andrew Rambaut, University of Edinburgh, Edinburgh, UK), exported as SVG files, and edited in Inkscape 1.3 (Inkscape Project, community-developed software).

The overall evolutionary divergence, as well as the intergroup (between different clades) and intragroup (within clades) evolutionary divergence between *Hepatozoon* spp. sequences were estimated using the p-distance method (gamma distribution = 1) in MEGA11 software [40]. Additionally, polymorphism metrics such as the number of polymorphic sites (S), the number of haplotypes (*H*), haplotype diversity (*Hd*), nucleotide diversity (π), and haplotype frequency among the sequences of this study were obtained using DnaSP 6.12.03 software (Julio Rozas, Universitat de Barcelona, Barcelona, Spain) [45]. The analyses excluded gaps and considered invariant sites. Subsequently, to determine the haplotype frequency of *Hepatozoon* spp. in rodents from Brazil and Chile, a haplotype network was constructed with sequences of 519 bp using the median-joining (MJ) method in PopART v.1.7 software (Leigh & Bryant, School of Biological Sciences, University of Auckland, Auckland, New Zealand). Analysis of molecular variance (AMOVA) [46] and the fixation index (Fst) [47] were calculated for the sequences from Brazil and Chile using the Arlequin software package, version 3.5.2.2 (Laurent Excoffier and Stéphane Lischer, University of Bern, Bern, Switzerland) [48]. AMOVA was used to test genetic variability between and within these groups, and the fixation index was employed to measure levels of genetic differentiation between haplotypes in two countries (Brazil and Chile).

## 3. Results

### 3.1. Distribution of Rodents

A total of 289 rodents were analyzed, with this sample comprising fourteen species of wild rodents from the Cricetidae family and two synanthropic species from the Muridae family. A total of 134 specimens were collected in the state of Rio de Janeiro, with 25 from Iguaba Grande (IG) and 109 from Nova Friburgo (NF), while 155 specimens were collected in the state of Paraná, including 52 from Cruz Machado (CM), 48 from Lidianópolis (LD), and 55 from Ponta Grossa (PG). Ponta Grossa exhibited the highest rodent diversity, with ten different species in the sample, whereas Iguaba Grande had the lowest, with only one. Among the wild rodents, *Akodon montensis* was the most abundant species (*n* = 106), followed by *Oligoryzomys nigripes* (*n* = 40) and *Akodon cursor* (*n* = 29), while *Mus musculus* was the most abundant of the synanthropic species (*n* = 42) (Table 1).

### 3.2. Morphometric Analysis of Gametocytes of Hepatozoon spp.

**Figure 1:**
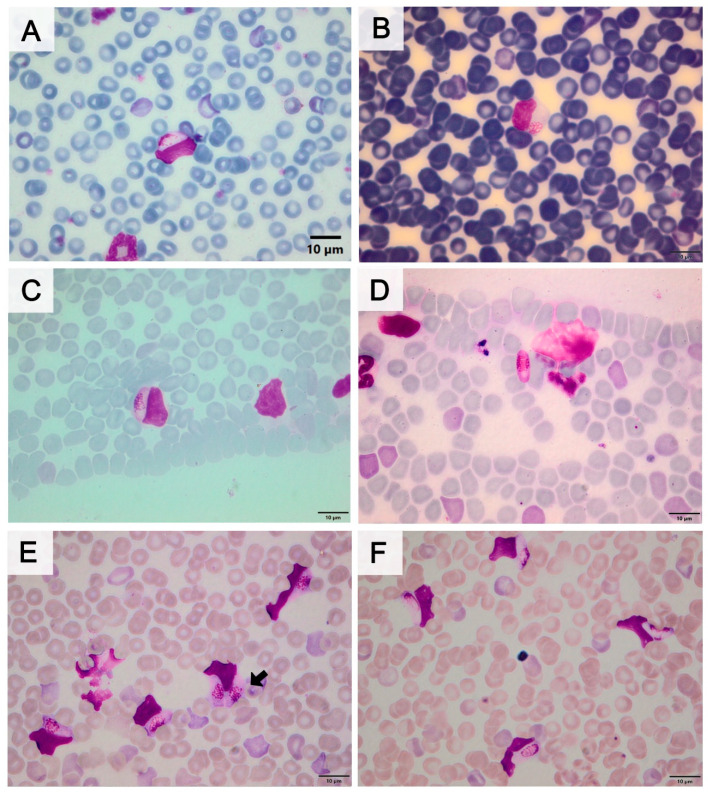
Gametocytes in rodent peripheral blood. (**A**). Gametocyte observed in a monocyte of *Oligoryzomys nigripes* (PQ807554, CM). (**B**). Gametocyte observed in a monocyte of *Akodon cursor* (PQ807526, NF). (**C**). Gametocyte observed in a monocyte of *Akodon montensis* (PQ807563, PG). (**D**). Free gametocyte observed in blood smear from *A. montensis* (PQ807542, CM). (**E**). Gametocyte observed in monocytes of *A. montensis* (PQ807538, CM). The black arrow indicates two gametocytes parasitizing the same cell. (**F**). Four gametocytes observed in monocytes of *A. montensis* blood smear (PQ807537, CM). Scale bar: 10 µm.

Gametocytes of *Hepatozoon* spp. were detected in blood smears from 10.72% of the sampled rodents (*n* = 31/289), with the positive rodents belonging to the species *A. cursor* (*n* = 2, NF), *A. montensis* (*n* = 28: two individuals from PG, nine from NF, and 17 from CM), and *O. nigripes* (*n* = 1, CM). The gametocytes exhibited an elongated and ovoid shape and light blue coloration and were located either inside leukocytes or free in the bloodstream. Their nuclei were slightly condensed and granular in appearance and were positioned either centrally or at one of the extremities. A case of double infection within a leukocyte was observed in the cytological analysis of an *A. montensis* specimen from CM (Figure 1).

The morphometric measurements of *Hepatozoon* gametocytes and their nuclei obtained in this study were compared with data previously reported for rodent hosts in other geographic regions (Table S2). In this study, capsule lengths of *Hepatozoon* spp. found in *Akodon* spp. and *Oligoryzomys nigripes* ranged from 10.13 µm to 10.95 µm, while widths varied between 4.40 µm and 4.77 µm. Nuclear lengths ranged from 5.83 µm to 6.54 µm, and nuclear widths ranged from 2.96 µm to 3.64 µm. The gametocytes detected in *A. montensis* specimens from the three localities (CM, NF, and PG) exhibited capsule measurements that were within a narrow range (10.23–10.95 µm in length; 4.44–4.69 µm in width), as did their nuclear dimensions. Overall, the values obtained in this study fall within the range of variation previously reported for rodent-associated *Hepatozoon* species in Brazil and Canada (Table S2).

The morphometric analysis of *A. montensis* gametocytes revealed that those observed in specimens from CM were significantly larger than those from NF (*p* < 0.05) and PG (*p* < 0.01). The nuclear lengths of gametocytes from PG were significantly greater than those from CM (*p* < 0.05) and NF (*p* < 0.01), and significant differences were also observed in the mean nuclear widths between gametocytes from CM and NF (*p* < 0.05), CM and PG (*p* < 0.01), and NF and PG (*p* < 0.01) (Table S3).

### 3.3. Detection and Phylogenetic Analysis of Hepatozoon spp.

In the PCR assay for the detection of *Hepatozoon* spp., 72 out of the 289 analyzed samples originating from seven species (Table 1) yielded an amplified fragment (~600 bp) of *Hepatozoon* sp. 18S rDNA (24.91%). These positive samples were from rodents collected in PG (4/55, 7.27%), NF (36/109, 33%), CM (30/52, 57.69%), and LD (2/48, 4.16%). The localities with the highest prevalence rates were CM (57.69%) and NF (33%), while samples from LD had the lowest prevalence (4.16%). *Akodon montensis* was the rodent species with the greatest number of positive individuals (46.23%, 49/106) (Table 1).

From the 72 *Hepatozoon* spp. amplifications, 41 sequences were obtained, including 31 from animals in which gametocytes had been detected in blood smears and 10 from animals with negative cytological analysis. The sequences from this study were deposited in GenBank under accession number PQ807526-66.

The BLASTn analysis of the representative haplotype sequences from this study displayed similarity to sequences previously obtained from rodents in Brazil, with identity values ranging from 99.81% to 100% (Table 2).

**Figure 2:**
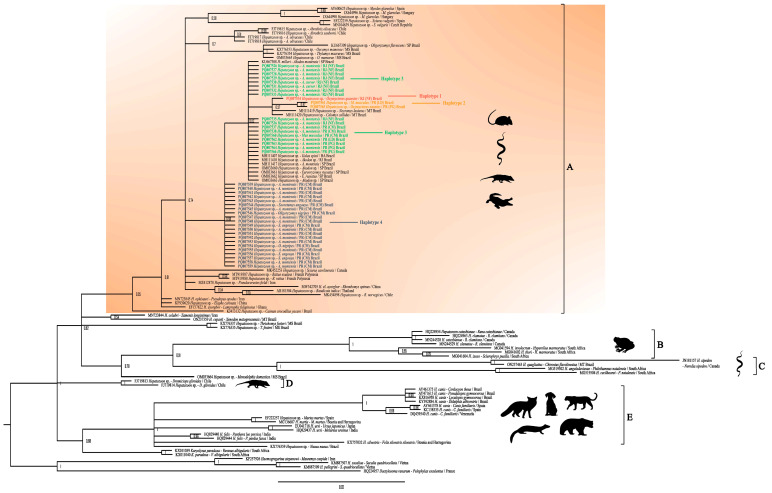
Phylogenetic relationships among *Hepatozoon* spp. based on 519 bp of 18S rDNA. The phylogenetic tree was inferred using Bayesian inference (BI 1) and the GTR + G + I model with 10,000,000 generations. Sequences detected in this study are highlighted in bold. *Dactylosoma ranarum* was used as an outgroup. The sequences obtained in this study clustered within Clade A, forming a subclade with other sequences previously obtained from rodents in Brazil (pp = 0.98), including the representative sequence of *H. milleri* (KU667308). The polymorphism analysis of the 41 *Hepatozoon* spp. sequences (519 bp) obtained in this study revealed the following values: *S* = 3, *Hd* = 0.577, π = 0.00151; and four haplotypes: Hap 1 (*n* = 1), Hap 2 (*n* = 2), Hap 3 (*n* = 17), and Hap 4 (*n* = 21). The colors of each haplotype are the same as those used in Figure 3, indicating their respective areas of occurrence. The frequency of the different haplotypes in the studied areas is also presented in Figure 3. The overall evolutionary divergence was 3%, while the genetic variability between Clade A and the other clades was 5% for Clades B and E, 6% for Clade C, and 3% for Clade D.

The BI analyses yielded ESS values greater than 200, demonstrating the robustness of the sampling. In the phylogenetic tree reconstructed with 114 partial 18S rDNA sequences (BI 1), the 108 *Hepatozoon* spp. sequences were grouped into five clades: Clade A—*Hepatozoon* spp. from rodents and reptiles from various regions worldwide, as well as from a marsupial in Brazil (KX776354) (posterior probability (pp) = 0.92); Clade B—*Hepatozoon* spp. from amphibians (pp = 1); Clade C—*Hepatozoon* spp. from snakes (pp = 1); Clade D—*Hepatozoon* spp. from marsupials in Chile (pp = 1); and Clade E—*Hepatozoon* spp. from carnivores (pp = 1) (Figure 2).

**Figure 3:**
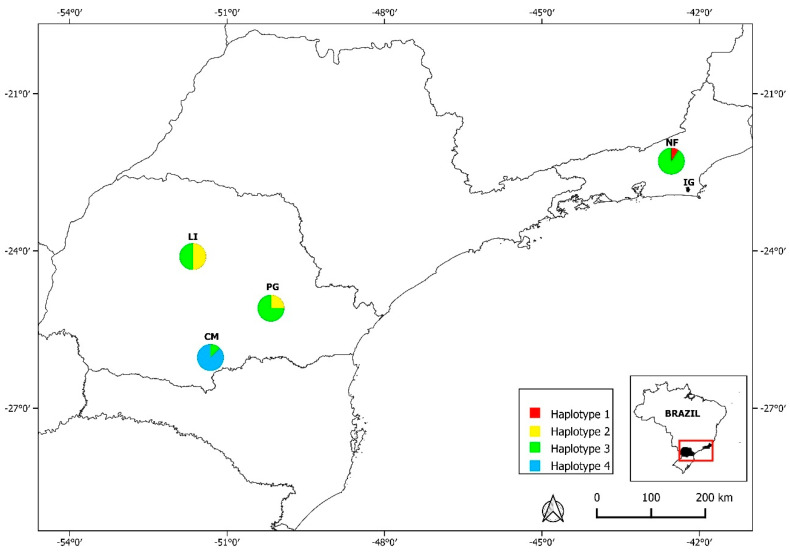
Map of part of the southern and southeastern regions of Brazil showing the frequency of *Hepatozoon* spp. haplotypes detected in rodents. The haplotype graphs were laid out over the areas sampled in the states of Paraná (LI, CM, and PG) and Rio de Janeiro (IG and NF). Abbreviations: CM, Cruz Machado; IG, Iguaba Grande; LI, Lidianópolis; NF, Nova Friburgo; PG, Ponta Grossa. This Map was created using QGIS version 3.42.0 [https://qgis.org/ (accessed on 14 March 2025)].

The second BI (2) analysis, conducted with 37 fragments of 1605 bp, revealed a tree topology similar to the previous analysis. The representative sequences of haplotypes 1, 3, and 4 from this study clustered closely with sequences obtained from rodents in Brazil (OM033660–OM033663), with strong statistical support (pp = 1) (Figure S1). It was not possible to obtain a long sequence for haplotype 2. In this analysis, the overall genetic distance was 4%, while the genetic distance between Clade A and the other clades was 6% for Clade B, 5% for Clades C and E, and 3% for Clade D.

**Figure 4:**
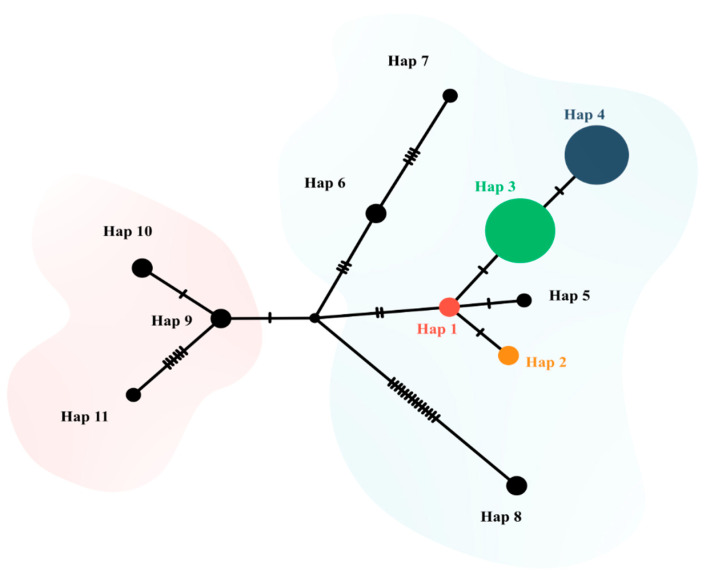
Haplotype network formed from 18S rDNA sequences (519 bp) of *Hepatozoon* spp. detected in small rodents from Brazil (haplotypes 1–8, light blue background) and Chile (haplotypes 9–11, light pink background). The size of the circles varies according to the number of sequences belonging to each haplotype. Mutational events are represented with vertical dashes. The haplotypes found in this study (1–4) are colored.

The haplotype network was constructed using the sequences from this study (*n* = 41) along with 20 sequences obtained in GenBank from rodents in Brazil (*n* = 15) and Chile (*n* = 5) (Figure 4, Table S4). The genetic diversity analysis of these 61 sequences yielded *S* = 32, *Hd* = 0.718, and *π* = 0.00641, and identified 11 haplotypes. The haplotypes analyzed formed four haplogroups originating from an ancestral sequence (not sampled) (Figure 4).

Based on haplotype formation, the gametocyte length of haplotype 4 (*n* = 153, mean = 10.951, SD = 0.726) was statistically greater than that of haplotype 3 (*n* = 96, mean = 10.682, SD = 0.817) (*p* < 0.01) (Table 3). No gametocytes were detected in samples of haplotypes 1 and 2. The genetic variation between countries was 56.78% (Fst = 0.567; *p* < 0.01), and the genetic variation within countries was 43.22%. Fst values revealed significant genetic differences between countries (Fst = 0.567; *p* < 0.01).

## 4. Discussion

This study detected *Hepatozoon* spp. infection in liver samples from six species of wild rodents from the Atlantic Forest biome and one synanthropic species in southern and southeastern regions of Brazil, specifically the states of Paraná and Rio de Janeiro. This is the first report of *Oxymycterus nasutus* and *Oxymycterus quaestor* as hosts of *Hepatozoon* spp. in Brazil and the first description of *Hepatozoon* spp. infection in wild rodents in Paraná.

Currently, few studies employ cytological analysis of blood smear slides as a diagnostic tool for *Hepatozoon* sp. and for the characterization of its gametocytes in animals [1,6,23,25,49,50]. When comparing the mean measurements of *Hepatozoon* sp. gametocytes (capsules and nuclei) obtained in this study with those reported in the literature, we found morphometric similarities to *Hepatozoon milleri* as described by Demoner et al. [6] in *Akodon montensis,* and to *Hepatozoon griseisciuri* as described by Leveille et al. [50] in the squirrel *Sciurus carolinensis*, although *S. carolinensis* does not occur in Brazil [50] (Table S2). The morphological analysis did not reveal any unique characteristics that could differentiate the gametocytes observed in this study.

Two gametocytes were observed within the same leukocyte in a specimen of *A. montensis* from CM (Figure 1). This finding, referred to as “double infection,” is commonly detected in reptiles parasitized by *Hepatozoon* spp. [51]. The presence of multiple *Hepatozoon* spp. gametocytes infecting the same leukocytes has been reported in cases of massive infections in snakes [2]. Based on this, we infer that a similar phenomenon may have occurred in the *A. montensis* specimen from CM, which could explain our observations. No reports of *Hepatozoon* sp. double infection within the same leukocyte in a rodent were found in the literature.

The morphometric analyses in this study revealed significant differences among the *A. montensis* gametocytes captured in CM, NF, and PG in terms of the gametocyte length and the nuclear length and width (Table S3). Similar findings have not been previously reported in the literature; however, based on these results and the detection of haplotypes, we compared the mean gametocyte measurements of those haplotypes for which the greatest numbers of gametocytes were analyzed. We observed that the mean gametocyte length of haplotype 4 (*n* = 153 gametocytes) was significantly greater than that of haplotype 3 (*n* = 96 gametocytes) (*p* < 0.01).

The PCR results demonstrated an overall prevalence of *Hepatozoon* spp. infection of 24.91% (72/289) in the analyzed liver samples, with *A. montensis* being the rodent species with the highest prevalence of infection (46.23%, 49/106), as well as the most abundant species in this study. This rodent species has previously been reported as being infected with *Hepatozoon* spp. [17,19] and identified as a host of *H. milleri* [6] in prior studies conducted in Brazil. The second-highest prevalence was detected in *Sooretamys angoyua* (45.45%, 5/11) in CM. This wild rodent species was previously reported to be infected with *Hepatozoon* sp. in São Paulo [13].

The prevalence detected in CM (*A. montensis*, *M. musculus*, *O. nigripes*, and *S. angouya*) was similar to the rate of positive tests for *Hepatozoon* spp. reported in the Botucatu region, São Paulo State (55.2% in *Akodon* sp., *Necromys lasiurus, Oligoryzomys flavescens, O. nigripes,* and *S. angouya*) [13]. It was also higher than the rate of positive tests (21.8% in *Thrichomys fosteri* and *Oecomys mamorae*) in the Pantanal region, Mato Grosso do Sul State [16], and rate of positive tests in *Calomys callosus* rodents in Mato Grosso State (7.1%) [14]. However, the highest prevalence found in this study was lower than that reported in Valdivia, Chile, where 82.43% of rodents (*Abrothrix longipilis*, *A. olivaceus*, *M. musculus*, *Oligoryzomys longicaudatus*, *Rattus norvegicus*, and *R. rattus*) were infected with *Hepatozoon* spp. [21].

A low prevalence (7.27%, 4/55) was detected in the municipality of PG, where the highest diversity (10 species) of wild rodents was found. This may be attributed to the fact that it is an area less impacted by humans, in contrast to the findings of Weck et al. [19], who detected *Hepatozoon* spp. infection only in areas with a high diversity of small mammals and ticks. Their findings suggest that the parasite’s life cycle occurs through a complex interaction between vertebrates and invertebrates.

Rodents of the species *A. montensis* showed the highest overall prevalence of infection compared to other rodent species and were found to be infected with *Hepatozoon* spp. in all areas where they were present, with significant prevalence values, which suggests that *A. montensis* may serve as a common intermediate host for *Hepatozoon* spp. in the Brazilian Atlantic Forest. This association may also reflect a possible parasite–host co-evolution event, as observed by Santodomingo et al. [22] in Chile, bearing in mind that most parasite species co-evolve with only a single host species [52]. The second-highest prevalence was detected in *Sooretamys angoyua* in CM. This wild rodent species was previously reported to be infected with *Hepatozoon* sp. in São Paulo [13].

BLASTn analyses revealed a close similarity (99.81% to 100%) between the *Hepatozoon* spp. sequences found in this study and sequences obtained from *Akodon* sp. and *Euryoryzomys russatus* rodents from the Atlantic Forest in the state of São Paulo [19]. Unfortunately, the lack of some species-level identification of the rodents prevents direct comparisons, particularly due to the occurrence of sympatry among *Akodon* species in that area [53]. The BLASTn analysis with the lowest similarity was observed for the sequences representing haplotype 1, which exhibited 99.81% similarity with the best BLAST hit sequence obtained from *Hepatozoon* sp. infecting *Akodon* sp. and *Euryoryzomys russatus* (OM033660–OM033663) from the state of São Paulo [19].

The sequences from this study are included in Clade A of *Hepatozoon* spp., which comprises sequences obtained from rodents and reptiles from different parts of the world, as well as from marsupials in the Americas. This clade remains distinct from Clade E, which includes sequences obtained from carnivores, a pattern also observed in previous studies [10,17,19]. A genetic divergence of 5% was observed between the sequences of these clades.

Our analyses support the association of *Hepatozoon* spp. parasitism between nonflying small mammals and reptiles, confirming the existence of an enzootic cycle in which the former act as intermediate or paratenic hosts, being preyed upon by reptiles within the trophic network. This hypothesis was experimentally demonstrated by Sloboda et al. [54], who successfully infected the snake *Python regius* with *Hepatozoon ayorgbor* through the ingestion of infected rodent tissues. However, some *Hepatozoon* species recovered from snakes remained isolated within Clade C, with a genetic distance of 6% (BI 1) to 5% (BI 2), suggesting that not all species of this parasite participate in the rodent-reptile enzootic cycle. Another possible route for the transmission of this parasitism could be the accidental ingestion of the definitive host by the reptile, as inferred for the snake *Xenodon matogrossensis*, which was found to be infected with *Hepatozoon cepavii* (ON237359) despite not preying on rodents [55]. We suggest that further studies on this parasitism involving these vertebrate orders should be encouraged to expand knowledge on these trophic relationships, which influence the transmission dynamics of *Hepatozoon* spp.

The *Hepatozoon* spp. sequences detected in this study correspond to four haplotypes. Haplotype 1 was identified in the mountainous region of the Atlantic Forest in the state of Rio de Janeiro in a specimen of *Oxymycterus quaestor* (PQ807534); however, it had previously been described in *Calomys callidus* rodents (MH111420) from the state of Mato Grosso. The circulation of the same *Hepatozoon* sp. haplotype among rodents from the Central–West and coastal regions of Brazil was reported in a previous study [17].

Although these two ecoregions have highly diverse landscapes, a recent study described the potential gene flow of another rodent species, *Necromys lasiurus*, occurring between the Atlantic Forest in Rio de Janeiro and the arid region of South America, including the state of Mato Grosso, since the start of the Holocene. As hosts migrate, their associated parasites circulate with them [56]. Infection by *Hepatozoon* sp. has been reported in a specimen of *N. lasiurus* [17] from Mato Grosso, whose sequence (MH111419) is a representative of haplotype 5, circulating among rodents in Brazil (Figure 4).

Haplotype 3 was previously described in rodents from the Atlantic Forest biome in the state of São Paulo, including in *Akodon* sp., *A. montensis*, and *Euryoryzomys russatus* [6,19], as well as in *Galea spixii* from the state of Bahia [17]. In this study, it was detected in *A. cursor* and *A. montensis* in the mountainous region of Rio de Janeiro (NF), as well as in *A. montensis* and *Mus musculus* in the three sampled municipalities in the state of Paraná (CM, LD, and PG). This was the most widespread haplotype, being detected in all analyzed areas where the parasite was present. In the phylogenetic tree reconstructed using the shorter 18S rDNA sequences, it demonstrated a close relationship with the representative sequence of *H. milleri* (KU667308). However, haplotypes 2 and 4 grouped separately, potentially representing new *Hepatozoon* spp. haplotypes present in rodents from the Atlantic Forest of Paraná. Haplotype 2 was detected in *Oxymycterus nasutus* and *M. musculus* in the municipalities of PG and LD, respectively, while haplotype 4 was found in *A. montensis*, *O. nigripes*, and *Sooretamys angouya* in CM. Finally, this study detected two distinct *Hepatozoon* spp. haplotypes infecting rodents, with varying prevalences across the analyzed municipalities (Figure 3). The discovery of new haplotypes is extremely valuable for filling gaps in the epidemiology of this parasite and may support the hypothesis of high genetic diversity of *Hepatozoon* spp. among wild rodent populations in Brazil [17,57].

In this study, two distinct haplotypes were detected in rodents of the species *A. montensis* (haplotypes 3 and 4) and *M. musculus* (haplotypes 2 and 3). The detection of different haplotypes within the same rodent species has been previously reported in Brazil [17] and Chile [21], but it was not possible to detect any association between a specific haplotype and a particular rodent group (wild or synanthropic), as was observed in Chile [21,22].

The genetic distance based on 18S rDNA among the sequences within Clade A (intragroup), which included our sequences, was very small (1%) and was insufficient to infer possible distinctions among parasite species. This was observed despite the inclusion of two of the three most hypervariable regions (V2 and V4) of the *Hepatozoon* spp. 18S rDNA gene in the 1600 bp sequences analyzed.

The nucleotide polymorphism analysis of *Hepatozoon* spp. sequences from this study revealed moderate haplotype diversity (*Hd* = 0.577)—higher than that reported in a study using sequences obtained from rodents across various Brazilian biomes (*Hd* = 0.426 [17]. However, it was lower than the diversity detected in rodents from Chile (*Hd* = 0.933 [21]. When analyzing the sequences from Brazil and Chile included in the haplotype network (*n* = 61), we observed high haplotype diversity (*Hd* = 0.701). In the haplotype network, sequences obtained from rodents in Brazil and Chile clustered into four haplogroups, all originating from an ancestral haplotype that has not yet been described (represented by the black circle in the center). The haplogroups were not shared between the two countries, with haplotypes 1 to 8 being detected in Brazil and haplotypes 9 to 11 being detected in Chile, suggesting a geographic structure [58]. These findings were supported by significant AMOVA and Fst results, which revealed genetic differences between the two countries; however, there is a gap in information between these sampled regions.

According to Ferreri et al. [58], the frequency, distribution, and arrangement of haplotypes can reveal a history of phylogeographic events related to coalescence theory. Undoubtedly, additional *Hepatozoon* spp. haplotypes exist in these countries; however, some of the detected sequences were not included in our analysis because they did not meet the size criterion (>500 bp) or corresponded to different regions of the 18S rDNA fragment.

The present study detected significant differences in the average sizes of gametocytes observed in rodents, establishing for the first time a significant relationship between *Hepatozoon* spp. haplotype and gametocyte length (*p* < 0.01). This reinforces the importance of optical microscopy as a complementary tool in molecular studies. In addition, two new hosts for this parasite among Brazilian wild rodents are described, namely *Oxymycterus nasutus* and *Oxymycterus quaestor,* and two novel *Hepatozoon* spp. haplotypes were detected among Brazilian rodents, i.e., haplotype 2 (*O. nasutus* and *M. musculus*) and haplotype 4 (*A. montensis, O. nigripes*, and *S. angouya*). Based on the haplotype network analysis, we suggest that there is geographic structuring among *Hepatozoon* haplogroups associated with rodents from Brazil and Chile (Fst = 0.567; *p* < 0.01). The application of additional molecular markers, including mitochondrial [36,50] and nuclear genes [59], may contribute to a more precise characterization of species and a deeper understanding of the diversity and evolution of parasitism within the order Rodentia.

## Figures and Tables

**Table 1:** Prevalence of *Hepatozoon* spp. according to host rodent species per municipality studied and total prevalence according to rodent species (last column) and municipality where capture occurred (last row).

Family		Study Areas					
Cricetidae		PG	CM	LD	IG	NF	*n*/Total (Prevalence)
	*Akodon cursor* (Winge, 1887)					6/29 (20.68%)	6/29 (20.68%)
	*Akodon montensis* (Tomas, 1913)	3/5 (60%)	18/26 (69.23%)	1/30 (3.33%)		27/45 (60%)	49/106 (46.23%)
	*Akodon paranaensis* (Christoff et al., 2000)	0/11					0/11
	*Delomys sublineatus* (Thomas, 1903)					0/1	0/1
	*Euryoryzomys russatus* (Wagner, 1848)	0/5					0/5
	*Juliomys ossitenuis* (Costa et al., 2007)	0/1					0/1
	*Necromys lasiurus* (Lund, 1841)	0/2		0/1			0/3
	*Nectomys squamipes* (Brants, 1827)	0/1					0/1
	*Oligoryzomys flavescens* (Waterhouse, 1837)	0/4					0/4
	*Oligoryzomys nigripes* (Olfers, 1818)	0/18	6/12 (50%)	0/1		0/9	6/40 (15%)
	*Oxymycterus nasutus* (Waterhouse, 1837)	1/6 (16.66%)					1/6 (16.66%)
	*Oxymycterus quaestor* (Thomas, 1903)					3/16 (18.57%)	3/16 (18.75%)
	*Sooretamys angouya* (Fischer, 1814)		5/11 (45.45%)			0/1	5/12 (38.46%)
	*Thaptomys nigrita* (Lichtenstein, 1829)	0/2					0/2
**Muridae**							
	*Mus musculus* (Linnaeus, 1758)		1/1 (100%)	1/16 (6.25%)	0/25		2/42 (4.76%)
	*Rattus rattus* (Linnaeus, 1758)		0/2			0/8	0/10
	*n*/total (prevalence)	4/55 (7.27%)	30/52 (57.69%)	2/48 (4.16%)	0/25	36/109 (33%)	72/289 (24.91%)

Abbreviations: PG, Ponta Grossa; CM, Cruz Machado; LD, Lidianópolis; IG, Iguaba Grande; NF, Nova Friburgo; *n*, number of infected rodents.

**Table 2:** Results of BLASTn analysis of the sequences of *Hepatozoon* spp. haplotypes found in rodents in this study.

Haplotype	GenBank ID	Host/Location (This Study)	Best Blast Hit ID	Host/Location (Best BLAST Hit)	Identity (%)	Query Coverage (%)	E-Value
Hap 1	PQ807534	*O. quaestor*/RJ	OM033660 e OM033663	*Akodon* sp., SP	99.81	100	0.0
			OM033661 e OM033662	*E. russatus*, SP			
Hap 2	PQ807561, PQ807565	*M. musculus*, *O. nasutus*/PR	MH111420	*Calomys callidus*, MT	99.84	100	0.0
Hap 3	PQ807530	*A. cursor/*RJ	OM033663, *Akodon* sp., SP	*Akodon* sp., SP	100	100	0.0
			OM033660, *Akodon* sp., SP	*Akodon* sp., SP			
Hap 4	PQ807539	*A. montensis/*PR	OM033660 e OM033663	*Akodon* sp., SP	99.94	100	0.0
			OM033661 e OM033662	*E. russatus*, SP			

Abbreviations: MT, State of Mato Grosso; PR, State of Paraná; RJ, State of Rio de Janeiro; SP, State of São Paulo.

**Table 3:** Average measurements of gametocyte length and width, and nucleus length and width of *Hepatozoon* spp. according to the haplotypes detected in this study.

Haplotypes	*n*	Mean (µM)		SD (µM)	Limit Values		
					Minimum (µM)	Maximum (µM)	*p*-Value
Gametocyte length							
Hap 3	96	10.82	a	0.82	8.12	12.26	
Hap 4	153	10.95	a	0.73	8.90	13.91	<0.01
Gametocyte width							
Hap 3	96	4.59	a	0.51	3.33	5.65	
Hap 4	153	4.55	a	0.46	3.29	5.86	0.560
Nuclear length							
Hap 3	96	6.05	a	1.14	3.76	8.72	
Hap 4	153	5.94	a	0.92	3.16	8.98	0.584
Nuclear width							
Hap 3	96	3.49	a	0.66	1.56	5.77	
Hap 4	153	3.37	b	0.51	2.19	4.72	0.109

Abbreviations: *n*, number of analyzed gametocytes; SD, standard deviation. Values followed by different letters in the same column differ significantly according to Tukey’s test. Haplotypes 1 and 2 did not have the minimum number of gametocytes required for statistical analysis.

## Data Availability

The datasets supporting the conclusions of this article are included within the article.

## Supplementary Materials

The following supporting information can be downloaded at: https://www.mdpi.com/article/10.3390/pathogens14080756/s1, Table S1: List of 18S rDNA sequences used in the phylogenetic analysis of *Hepatozoon* spp. The first column shows the GenBank accession, followed by the identification of the parasite, host species, host order, and country of origin. Table S2: Morphometric data on the capsule and nucleus of different *Hepatozoon* species detected in rodents found in the literature and in this study. Table S3: Average measurements of gametocyte length and width and nucleus length and width of *Hepatozoon* spp. observed in rodents in this study, according to locality. Table S4: List of *Hepatozoon* spp. haplotypes used in the haplotype network (Figure 4), relating the respective haplotype to the GenBank accession, intermediate host, and country of origin. Figure S1: Phylogenetic relationship among *Hepatozoon* spp. based on a final dataset of 1605 bp sequences of 18S rDNA. The phylogenetic tree was inferred using Bayesian inference (BI 2) and the GTR + G + I model with 10,000,000 generations. Sequences detected in this study are highlighted in bold. *Dactylosoma kermiti* was used as an outgroup.

## Abbreviations

The following abbreviations are used in this manuscript:

DNA	Deoxyribonucleic acid
PCR	Polymerase chain reaction
Cytb	Cytochrome b
µM	Micrometer
SD	Standard deviation
