# Supplementary material for: Morphometric and Molecular Insights into Hepatozoon spp. in Wild and Synanthropic Rodents from Southern and Southeastern Brazil
Source: Pathogens. 2025 Jul 31;14(8):756. doi: 10.3390/pathogens14080756 (PMC12389497; doi:10.3390/pathogens14080756)
Supplement: Supplementary file 1 [file pathogens-14-00756-s001.zip › pathogens-3637838-supplementary.pdf]

Supplementary materials

Table S1: List of 18S rDNA sequences used in the phylogenetic analysis of *Hepatozoon* spp. The first column shows the GenBank accession, followed by the identification of the parasite, host species, host order, and country of origin.

GenBank ID	Parasite	Host specie	Order	Country
AB181504	*Hepatozoon* sp.	*Bandicota indica*	Rodentia	Thailand
AY461375	*Hepatozoon canis*	*Dusicyon thous*	Carnivora	Brazil
AY461378	*Hepatozoon canis*	*Canis familiaris*	Carnivora	Spain
AY471615	*Hepatozoon canis*	*Pseudalopex gymnocercus*	Carnivora	Brazil
AY600625	*Hepatozoon* sp.	*Myodes glareolus*	Rodentia	Spain
DQ439540	*Hepatozoon canis*	*Canis familiaris*	Carnivora	Venezuela
EF157822	*Hepatozoon ayorgbor*	*Lamprophis fuliginosus*	Squamata	Ghana
EF222257	*Hepatozoon* sp.	*Martes martes*	Carnivora	Spain
EF222259	*Hepatozoon* sp.	*Sciurus vulgaris*	Rodentia	Spain
EU041718	*Hepatozoon*	*Ursus thibetanus japonicus*	Carnivora	Japan
FJ719813	*Hepatozoon* sp.	*Dromiciops gliroides*	Marsupialia	Chile
FJ719814	*Hepatozoon* sp.	*Dromiciops gliroides*	Marsupialia	Chile
FJ719815	*Hepatozoon* sp.	*Abrothrix olivaceus*	Rodentia	Chile
FJ719816	*Hepatozoon* sp.	*Abrothrix sanborni*	Rodentia	Chile
FJ719817	*Hepatozoon* sp.	*Abrothrix olivaceus*	Rodentia	Chile
FJ719818	*Hepatozoon* sp.	*Abrothrix olivaceus*	Rodentia	Chile
HQ224954	*Hepatozoon catesbianae*	*Rana catesbeiana*	Anura	Canada
HQ224957	*Dactylosoma ranarum*	*Pelophylax esculentus*	Anura	France
HQ224963	*Hepatozoon clamatae*	*Rana clamitans*	Anura	Canada
HQ829437	*Hepatozoon ursi*	*Melursus ursinus*	Carnivora	India
HQ829440	*Hepatozoon felis*	*Panthera leo persica*	Carnivora	India
HQ829444	*Hepatozoon felis*	*Panthera pardus fusca*	Carnivora	India
JN181157	*Hepatozoon sipedon*	*Nerodia sipedon sipedon*	Squamata	Canada
JX644996	*Hepatozoon* sp.	*Myodes glareolus*	Rodentia	Hungary
JX644998	*Hepatozoon* sp.	*Myodes glareolus*	Rodentia	Hungary
KC138535	*Hepatozoon canis*	*Canis familiaris*	Carnivora	Spain
KF257926	*Haemogregarina stepanowi*	*Mauremys caspica*	Testudines	Iran
KF939620	*Hepatozoon* sp.	*Elaphe carinata*	Squamata	China
KJ413132	*Hepatozoon* sp.	*Caiman crocodilus yacare*	Crocodylia	Brazil
KM887507	*Haemogregarina sacaliae*	*Sacalia quadriocellata*	Testudines	Vietna
KM887509	*Haemogregarina pellegrini*	*Sacalia quadriocellata*	Testudines	Vietna
KU667308	*Hepatozoon milleri*	*Akodon montensis*	Rodentia	Brazil
KU667309	*Hepatozoon* sp.	*Oligoryzomys flavescens*	Rodentia	Brazil
KX011039	*Karyolysus paradoxa*	*Varanus albigularis*	Squamata	South Africa
KX011040	*Karyolysus paradoxa*	*Varanus albigularis*	Squamata	South Africa
KX757032	*Hepatozoon silvestris*	*Felis silvestris silvestris*	Carnivora	Bosnia and Herzegovina
KX776335	*Hepatozoon* sp.	*Thrichomys fosteri*	Rodentia	Brazil
KX776337	*Hepatozoon* sp.	*Thrichomys fosteri*	Rodentia	Brazil
KX776353	*Hepatozoon* sp.	*Oecomys mamorae*	Rodentia	Brazil
KX776354	*Hepatozoon* sp.	*Thylamys macrurus*	Marsupialia	Brazil
KX776359	*Hepatozoon* sp.	*Nasua nasua*	Marsupialia	Brazil
KX816958	*Hepatozoon canis*	*Lycalopex gymnocercus*	Carnivora	Brazil
KY392884	*Hepatozoon canis*	*Didelphis albiventris*	Marsupialia	Brazil
MG041594	*Hepatozoon involucrum*	*Hyperolius marmoratus*	Anura	South Africa
MG041602	*Hepatozoon thori*	*Hyperolius marmoratus*	Anura	South Africa
MG041604	*Hepatozoon ixoxo*	*Sclerophrys pusilla*	Anura	South Africa
MG136687	*Hepatozoon martis*	*Martes martes*	Carnivora	Bosnia and Herzegovina
MG519502	*Hepatozoon angeladaviesae*	*Philothamnus natalensis natalensis*	Squamata	South Africa
MG519504	*Hepatozoon cecilhoarei*	*Philothamnus natalensis natalensis*	Squamata	South Africa
MH111407	*Hepatozoon* sp.	*Galea spixii*	Rodentia	Brazil
MH111410	*Hepatozoon* sp.	*Akodon* sp.	Rodentia	Brazil

Table S1 (continuation): List of 18S rDNA sequences used in the phylogenetic analysis of *Hepatozoon* spp. The first column shows the GenBank accession, followed by the identification of the parasite, host species, host order, and country of origin.

GenBank ID	Parasite	Host specie	Order	Country
MH111417	*Hepatozoon* sp.	*Akodon montensis*	Rodentia	Brazil
MH111419	*Hepatozoon* sp.	*Necromys lasiurus*	Rodentia	Brazil
MH111420	*Hepatozoon* sp.	*Calomys callidus*	Rodentia	Brazil
MK452253	*Hepatozoon griseisciuri*	*Sciurus carolinensis*	Rodentia	Canada
MK454898	*Hepatozoon* sp.	*Rattus norvegicus*	Rodentia	Chile
MN104639	*Hepatozoon sciuri*	*Sciurus vulgaris*	Rodentia	Czech Republic
MN244528	*Hepatozoon catesbianae*	*Rana clamitans*	Anura	Canada
MN244529	*Hepatozoon clamatae*	*Rana clamitans*	Anura	Canada
MN723844	*Hepatozoon colubri*	*Zamenis longissimus*	Squamata	Iran
MN723845	*Hepatozoon ophisauri*	*Pseudopus apodus*	Squamata	Iran
MT919387	*Hepatozoon* sp.	*Rattus exulans*	Rodentia	French Polynesia
MT919388	*Hepatozoon* sp.	*Rattus rattus*	Rodentia	French Polynesia
MZ412878	*Hepatozoon* sp.	*Pseudocerastes fieldi*	Squamata	Iran
MW342705	*Hepatozoon* cf. *ayorgbor*	*Rhombomys opimus*	Rodentia	China
OM033660	*Hepatozoon* sp.	*Akodon sp.*	Rodentia	Brazil
OM033661	*Hepatozoon* sp.	*Euryoryzomys russatus*	Rodentia	Brazil
OM033662	*Hepatozoon* sp.	*Euryoryzomys russatus*	Rodentia	Brazil
OM033663	*Hepatozoon* sp.	*Akodon sp.*	Rodentia	Brazil
OM033664	*Hepatozoon* sp.	*Monodelphis domestica*	Marsupialia	Brazil
OM033665	*Hepatozoon* sp.	*Oecomys mamorae*	Rodentia	Brazil
ON237359	*Hepatozoon cepavii*	*Xenodon matogrossensis*	Squamata	Brazil
ON237463	*Hepatozoon quagliattus*	*Chironius flavolineatus*	Squamata	Brazil

Table S2: Morphometric data on the capsule and nucleus of different *Hepatozoon* species detected in rodents found in the literature and in this study.

Specie parasite	Intermediate host	Country	Capsula (μM)*	Nuclei (μM)*	Reference
H. perniciosum	*Rattus norvegicus*	USA	12 x 6	-	[1]
H. akodoni	*Akodon fuliginosus*	Brazil	10 x 3.5	4 x 2.5	[25]
Hepatozoon sp.	*Sigmodon hispidus* e *Peromyscus leucopus*	USA	9.6 x 3.6	7.5 x 3.0	[23]
H. erhardovae	*Clethrionomys glareolus*	Poland	9.4 x 3.4	4.8 x 2.5	[49]
Hepatozoon sp.	*Microtus oeconomus*	Poland	9.4 x 3.3	4.8 x 2.4	[49]
H. milleri	*Akodon montensis*	Brazil	10.9 x 4.9	5.6 x 3.8	[6]
H. griseisciuri	*Sciurus carolinensis*	Canada	10.9 x 4.1	5.4 x 3.1	[50]
Hepatozoon sp.	*Akodon montensis* - CM	Brazil	10.95 x 4.52	5.89 x 3.44	This study
Hepatozoon sp.	*Akodon montensis* - NF	Brazil	10.65 x 4.69	5.92 x 3.64	This study
Hepatozoon sp.	*Akodon montensis* - PG	Brazil	10.23 x 4.44	6.49 x 2.96	This study
Hepatozoon sp.	*Akodon cursor* - NF	Brazil	10.49 x 4.77	5.83 x 3.59	This study
Hepatozoon sp.	*Oligoryzomys nigripes* - CM	Brazil	10.13 x 4.40	6.54 x 3.22	This study

* lenght x width average.

Table S3. Average measurements of gametocyte length and width and nucleus length and width of *Hepatozoon* spp. observed in rodents in this study, according to locality.

Study areas	N	Mean (µM)		SD (µM)	Limit values		p-value
					Minimum (µM)	Maximum (µM)	
Gametocyte length							
Cruz Machado	165	10.95	a	0.72	8.90	13.90	< 0.05
Nova Friburgo	45	10.65	b	0.71	8.69	12.05	< 0.01
Ponta Grossa	11	10.23	b	1.10	8.12	12.26	ns
Gametocyte width							
Cruz Machado	165	4.52	a	0.44	3.29	5.86	
Nova Friburgo	45	4.69	a	0.53	3.33	5.64	0.082
Ponta Grossa	11	4.44	a	0.59	3.56	5.55	
Nuclear length							
Cruz Machado	165	5.89	a	0.91	3.16	8.38	ns
Nova Friburgo	45	5.92	a	1.12	3.76	8.72	< 0.01
Ponta Grossa	11	6.50	b	0.95	4.12	8.01	< 0.05
Nuclear width							
Cruz Machado	165	3.44	a	0.44	2.41	4.72	< 0.05
Nova Friburgo	45	3.64	b	0.61	2.15	5.77	< 0.01
Ponta Grossa	11	2.96	c	0.72	1.92	4.29	< 0.01

Abbreviations: N, number of analyzed gametocytes; SD, standard deviation; ns, non-significant.
Values followed by different letters in the same column differ significantly by Tukey's test. Lidianópolis and Iguaba Grande did not have the minimum number of gametocytes required for statistical analysis.

Table S4: List of *Hepatozoon* spp. haplotypes used in the haplotype network (Figure 4), relating the respective haplotype to the GenBank accession, intermediate host, and country of origin.

Haplotype	GenBank number	Host	Country
Hap 1	PQ807534	*Oxymycterus quaestor*	Brazil
Hap 1	MH111420	*Calomys callidus*	Brazil
Hap 2	PQ807561	*Mus musculus*	Brazil
Hap 2	PQ807565	*Oxymycterus nasutus*	Brazil
Hap 3	PQ807526	*Akodon montensis*	Brazil
Hap 3	PQ807527	*Akodon montensis*	Brazil
Hap 3	PQ807528	*Akodon montensis*	Brazil
Hap 3	PQ807529	*Akodon montensis*	Brazil
Hap 3	PQ807530	*Akodon cursor*	Brazil
Hap 3	PQ807531	*Akodon cursor*	Brazil
Hap 3	PQ807532	*Akodon montensis*	Brazil
Hap 3	PQ807533	*Akodon montensis*	Brazil
Hap 3	PQ807535	*Akodon montensis*	Brazil
Hap 3	PQ807536	*Akodon montensis*	Brazil
Hap 3	PQ807537	*Akodon montensis*	Brazil
Hap 3	PQ807538	*Akodon montensis*	Brazil
Hap 3	PQ807560	*Mus musculus*	Brazil
Hap 3	PQ807562	*Akodon montensis*	Brazil
Hap 3	PQ807563	*Akodon montensis*	Brazil
Hap 3	PQ807564	*Akodon montensis*	Brazil
Hap 3	PQ807566	*Akodon montensis*	Brazil
Hap 3	KU667308	*Akodon montensis*	Brazil
Hap 3	MH111407	*Galea spixii*	Brazil
Hap 3	MH111410	*Akodon* sp.	Brazil
Hap 3	MH111417	*Akodon montensis*	Brazil
Hap 3	OM033660	*Akodon* sp.	Brazil
Hap 3	OM033661	*Euryoryzomys russatus*	Brazil
Hap 3	OM033662	*Euryoryzomys russatus*	Brazil
Hap 3	OM033663	*Akodon* sp.	Brazil
Hap 4	PQ807539	*Akodon montensis*	Brazil
Hap 4	PQ807540	*Akodon montensis*	Brazil
Hap 4	PQ807541	*Akodon montensis*	Brazil
Hap 4	PQ807542	*Akodon montensis*	Brazil
Hap 4	PQ807543	*Akodon montensis*	Brazil
Hap 4	PQ807544	*Sooretamys angouya*	Brazil
Hap 4	PQ807545	*Akodon montensis*	Brazil
Hap 4	PQ807546	*Oligoryzomys nigripes*	Brazil
Hap 4	PQ807547	*Akodon montensis*	Brazil
Hap 4	PQ807548	*Akodon montensis*	Brazil
Hap 4	PQ807549	*Sooretamys angouya*	Brazil
Hap 4	PQ807550	*Akodon montensis*	Brazil
Hap 4	PQ807551	*Akodon montensis*	Brazil
Hap 4	PQ807552	*Akodon montensis*	Brazil
Hap 4	PQ807553	*Akodon montensis*	Brazil
Hap 4	PQ807554	*Oligoryzomys nigripes*	Brazil
Hap 4	PQ807555	*Akodon montensis*	Brazil
Hap 4	PQ807556	*Sooretamys angouya*	Brazil
Hap 4	PQ807557	*Sooretamys angouya*	Brazil
Hap 4	PQ807558	*Akodon montensis*	Brazil
Hap 4	PQ807559	*Akodon montensis*	Brazil
Hap 5	MH111419	*Necromys lasiurus*	Brazil
Hap 6	OM033665	*Oecomys mamorae*	Brazil
Hap 6	KX777653	*Oecomys mamorae*	Brazil
Hap 7	KU667309	*Oligoryzomys flavescens*	Brazil
Hap 8	KX776335	*Thrichomys fosteri*	Brazil
Hap 8	KX776337	*Thrichomys fosteri*	Brazil
Hap 9	FJ719817	*Abrothrix olivaceus*	Chile
Hap 9	FJ719818	*Abrothrix olivaceus*	Chile
Hap 10	FJ719815	*Abrothrix olivaceus*	Chile
Hap 10	FJ719816	*Abrothrix sanborni*	Chile
Hap 11	MK454898	*Rattus norvegicus*	Chile

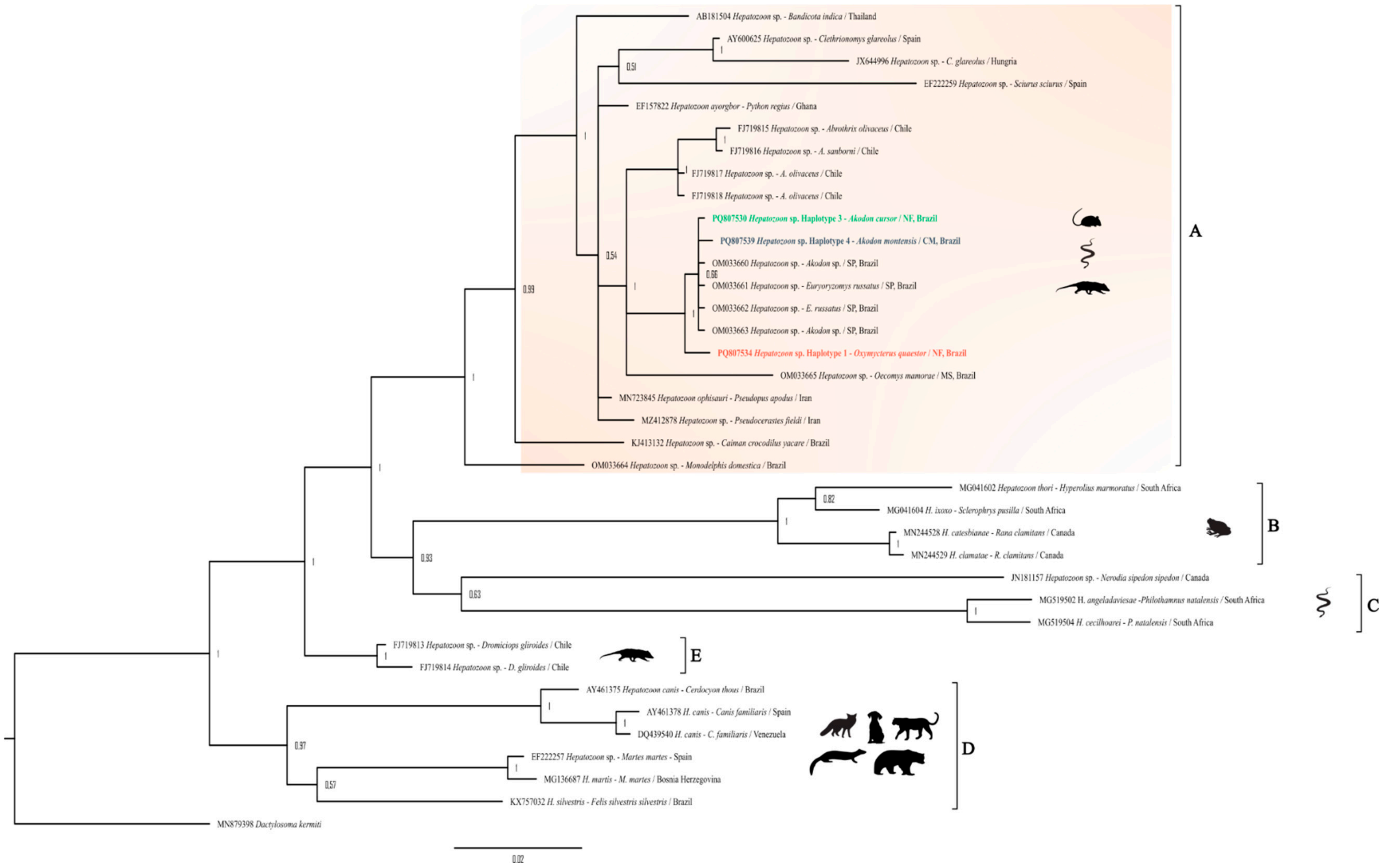

Figure S1. Phylogenetic relationship among *Hepatozoon* spp. based on a final dataset of 1605 bp sequences of 18S rDNA. The phylogenetic tree was inferred using Bayesian inference (BI 2) and the GTR + G + I model with 10,000,000 generations. Sequences detected in this study are highlighted in bold. *Dactylosoma kermiti* was used as an outgroup.
